# Editorial: Healthspan and neural aging: Merging the exposome and one environmental health - From molecular mechanisms to epidemiology

**DOI:** 10.3389/fnagi.2023.1172967

**Published:** 2023-03-14

**Authors:** Mary Ann Ottinger, Gaylia Jean Harry, Kristen Malecki, John Pierce Wise

**Affiliations:** ^1^Department of Biology and Biochemistry, University of Houston, Houston, TX, United States; ^2^Neurotoxicoogy Group, National Institute of Environmental Health, Durham, NC, United States; ^3^Division of Environmental and Occupational Health Sciences, School of Public Health, University of Illinois, Chicago, IL, United States; ^4^Wise Laboratory of Environmental and Genetic Toxicology, Department of Pharmacology and Toxicology, University of Louisville, Louisville, KY, United States

**Keywords:** health span, exposome, biology of aging, health disparities, one environmental health, gerontogens, aging and environmental chemicals

Aging begins during development and establishes a framework for later events. The process occurs through the lifespan and is influenced by individual genetics and life events. Such life events include stress and reflect a composite of multiple, interrelated, external factors including chemical and physical environments and modifying social factors. Genetic and life-style factors influence healthy aging and there is mounting evidence for short- and long-term effects of environmental factors on age-related health outcomes, such as cardiovascular disease, metabolic disorders, cancer, and neurodegenerative disorders. Alterations in key biological processes can diminish one's ability to metabolize, compensate, adapt, and recover from insults due to adverse stressors or health events. Such shifts can be associated with increased age-related biological susceptibility to physiological decline and disease and serve as a basis for considering the elderly as a vulnerable and sensitive population (Risher et al., 2010; Malecki et al.).

Environmental factors that can adversely affect the trajectory of healthy aging of an organism, gerontogens, have been formally considered since 1987 (Baker and Rogul, 1987; Martin, 1987). This framework has previously defined toxicology as it relates to aging (Sorrentino et al., 2014) however, complex environment factors can exacerbate or mitigate adverse outcomes over time (Geller and Zenick, 2005). Thus, it is essential to understand effects of gerontogens on aging processes and differential susceptibility of individuals across the lifespan (Wise). While there is limited ability to undo effects of earlier exposures, developing approaches to modify disease progression may be within our grasp, especially in the cases of age-related diseases (Alhasan et al.). The impact of social determinants and value of community engagement, social interactions, and the love of a pet are substantial and associated with a more positive aging trajectory (Nwanaji-Enwerem et al., 2021; Noren Hooten et al., 2022; McDonough et al.). The establishment of predictive and effective interventions will support these efforts and be facilitated by the establishment of biomarkers indicative of health related effects or susceptibility such as those for inflammation, somatic mutations, and epigenetic alterations (Knobel et al.). A significant contribution to our understanding of such relationships will come from the inclusion of biomarkers of exposure not only for environmental chemicals but also for stress. This is the rationale for the current consideration of the full array of environmental exposures over the lifespan, the exposome (Nwanaji-Enwerem et al., 2021; Kalia et al., 2022). The consideration of environmental stressors that encompass social and environmental factors are critical to determine contributions to an individual's aging (Figure 1). Research at the intersection of aging and the environment that consider social and environmental exposures wholistically can fill existing knowledge gaps on malleable features of aging, identify modifiable risk factors, effective therapeutic interventions, and provide a basis for regulatory and societal policies to promote healthy aging and limit population-based disparities in life-expectancy and chronic disease burden.

**Figure 1:**
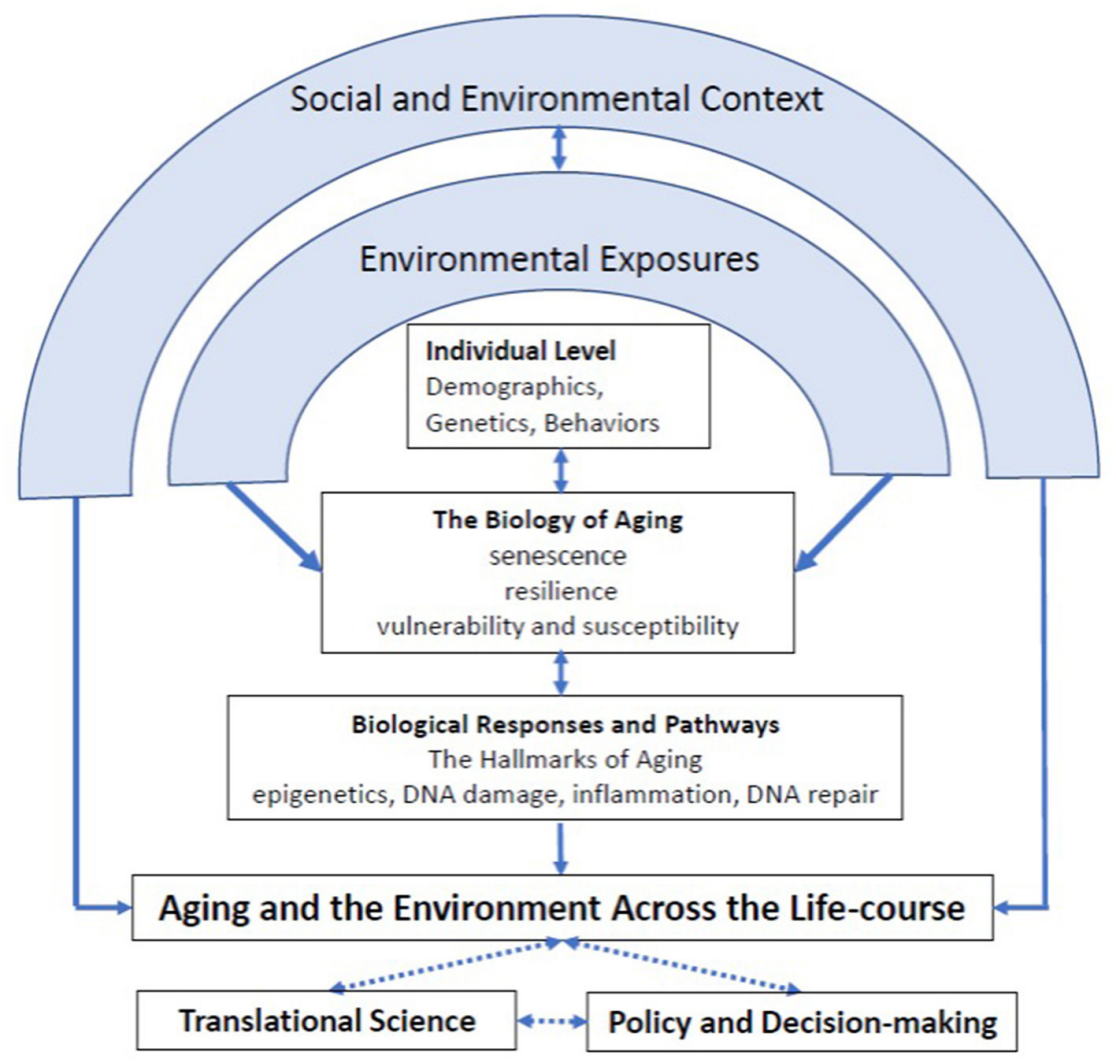
Integrating aging and environmental health research.

Human health is closely related to shared environments and the health of multiple organisms within these environments. Thus, relationships between environments and health are not limited to the human population but rather, can be observed across the spectrum of biological organisms (Comizzoli and Ottinger, 2021). Multi-disciplinary collaborations across ecological, biological, and health fields are critical to characterize the relationship between our personal environment and a healthy lifespan (Malecki et al.; National Academies of Science, 2020).

There are many facets to our interrelationship with our environment and their changing dynamics over the lifespan. Gaining an appreciation of these dynamics in response to complex chemical mixture exposures, pharmaceutical, societal, and lifestyle will contribute to our understanding of the intersections, unique vulnerabilities, and possible effective therapeutic interventions to promote health in the later stages of life (Ding et al., 2022; Kalia et al., 2022; Malecki et al.). Such information will provide a basis for regulatory and societal policies to promote a health lifespan (Geller and Zenick, 2005).

## Author contributions

All authors listed have made a substantial, direct, and intellectual contribution to the work and approved it for publication.

## References

[B1] Baker S. R. Rogul M. (1987). Environmental Toxicity and the Aging Process. New York, NY: Alan R. Liss.

[B2] Comizzoli P. Ottinger M. A. (2021). Understanding reproductive aging in wildlife to improve animal conservation and human reproductive health. Front. Cell Dev. Biol. 9, 680471. 10.3389/fcell.2021.680471 34095152 PMC8170016

[B3] Ding E. Wang Y. Liu J. Tang S. Shi X. (2022). A review on the application of the exposome paradigm to unveil the environmental determinants of age-related diseases. Hum. Genomics 16, 54. 10.1186/s40246-022-00428-6 36348440 PMC9644545

[B4] Geller A. M. Zenick H. (2005). Aging and the environment: a research framework. Environ. Health Perspect. 113, 1257–1262. 10.1289/ehp.7569 16140638 PMC1280412

[B5] Kalia V. Belsky D. W. Baccarelli A. A. Miller G. W. (2022). An exposomic framework to uncover environmental drivers of aging. Exposome 2, osac002. 10.1093/exposome/osac002 35295547 PMC8917275

[B6] Martin G. M. (1987). Interactions of aging and environmental agents: the gerontological perspective. Prog. Clin. Biol. Res. 228, 25–80. 3554264

[B7] NAS, National Academies of Science Integrating the Science of Aging Environmental Health Research, Proceedings of a Workshop – in Brief. (2020). Available online at: https://nap.nationalacademies.org/catalog/25908/integrating-the-science-of-aging-and-environmental-health-research-proceedings 32924387

[B8] Noren Hooten N. Pacheco N. L. Smith J. T. Evans M. K. (2022). The accelerated aging phenotype: The role of race and social determinants of health on aging. Ageing Res. Rev. 73, 101536. 10.1016/j.arr.2021.101536 34883202 PMC10862389

[B9] Nwanaji-Enwerem J. C. Jackson C. L. Ottinger M. A. Cardenas A. James K. A. Malecki K. M. C.. (2021). Adopting a “compound” Exposome approach in environmental aging biomarker research: a call to action for advancing racial health equity. Environ. Health Perspect. 129, 1–8. 10.1289/EHP8392 33822649 PMC8043128

[B10] Risher J. F. Todd G. D. Meyer D. Zunker C. L. (2010). The elderly as a sensitive population in environmental exposures: making the case. Rev. Environ. Contam. Toxicol. 207, 95–157. 10.1007/978-1-4419-6406-9_2 20652665

[B11] Sorrentino J. A. Sanoff H. K. Sharpless N. E. (2014). Defining the toxicology of aging. Trends Mol. Med. 20, 375–384. 10.1016/j.molmed.04~004 24880613 PMC4082749

